# Books and Authors

**Published:** 1911-05

**Authors:** 


**Modern Treatment**: The Management of Disease with Medicinal and Non-Medicinal Remedies. By Eminent American and English authorities. Edited by **Hobart Amory Hare**, M.D., Professor of Therapeutics and Materia Medica, Jefferson Medical College, Philadelphia, assisted by **H. R. M. Landis**, M.D., Medical director to the Phipps Institute for Tuberculosis and Physician to the White Haven Sanatorium. In two octavo volumes, Vols. I. and II. comprising 1,800 pages, with numerous engravings and full page plates. Lea and Febiger, Publishers, Philadelphia and New York. 1911. (Price per volume in cloth, $6.00; half morocco, $7.50, net.)

In the two volumes of this work already issued medical literature has been enriched by the excellence of the material and the manner in which it is presented. Hardly two months have passed since the publication of the first volume and its success was apparent from the beginning. When the first announcement was made a year ago that such a work was in course of preparation, under the direction of Hobart Amory Hare, it was expected that it would be in its completed form no less than a masterpiece; especially when the list of contributors was made public. The finished work bears out all expectations and the profession now has at its command these volumes, which will be found of great value for daily reference. Encyclopedic in character, it is in no sense an encyclopedia in the strictest interpretation of the term; rather is it the essence of modern therapeusis, presented in intelligent fashion and covering the entire field of present-day medicine. The first volume is practically a summary of all medical advances and is rich in advice. There are the general considerations; the treatment of disease by non-medicinal measures which, of course, include hydrotherapy, electrotherapeutics, *x*-ray and raido-actives, nutrition, rest and vaccine, serum and glandular therapy. The third section of this volume deals with infectious diseases.

The second volume, which has just been received, covers the treatment of parasitic, circulatory, digestive and respiratory diseases, those of nutrition and the diatheses, the skin, the eye, the ear, nervous system and genitourinary tract.

**A Treatise on Diseases of the Nose, Throat and Ear.** By **William Lincoln Ballenger**, M.D., Professor of Laryngology, Rhinology and Otology in the College of Physicians and Surgeons, Chicago. New (3d) edition, thoroughly revised. Octavo, 983 pages, with 506 engravings, mostly original, and 22 plates. Lea & Febiger, Philadelphia and New York. 1911. (Cloth, $5.50 net.)

This work has already attained a remarkable record of popularity. Three editions have been called for in less than three years, and during part of this time it has been entirely out of print, owing to the unexpected rapidity of exhaustion. Ballenger made adequate preparation for his treatise by adding to his library some 3,000 monographs, pamphlets and reprints, obtained in this country and Europe, thus possessing the very latest literature on the subjects dealt with. His work is most comprehensive, covering both the medical and surgical treatment of the whole region of the nose, throat and ear.

The book is illustrated most profusely, the engravings for the greater part having been made from original drawings. The successive steps of nearly every accepted operation are shown. The book as it now stands may be fairly regarded as the most modern exposition of diseases of the nose, throat and ear by any author, American or foreign.

**The Treatment of Disease.**..A manual of Practical Medicine. By **Reynold Webb Wilcox,** M.A., M.D., Professor of Medicine (retired), at the New York Post-Graduate Medical School and Hospital. Third edition. Octavo, pp. 1048. Philadelphia: P. Blakiston's Son & Co. 1911. (Cloth, $7.50.)

This is the third edition in four years and is thoroughly revised and improved except in those portions devoted to diseases of the genitourinary tract. There have been added to the previous editions forty-three sections devoted to as many diseases and all the newest methods and improved treatments of internal medicine have been given place in the book which is, with the exception mentioned, of exceptional value to the physician.

The treatment outlined for cystitis is not that which is recognised as best by those who specialise. The general practitioner should not blindly accept the suggestion that prostatectomy be advised in cases of hypertrophy unless he is certain that the hypertrophy is the only cause of obstruction. The recent advances in this field tend to the belief that colon vaccine administered will in a large number of cases relieve the patient and make operation unnecessary. The treatment outlined in both chronic and acute cystitis is generally inadequate. Few writers on internal medicine and treatment escape the pitfalls of urinary surgery and treatment, and in this instance the statement that the operation for stricture in chronic cystitis due to urethral obstruction is easy is merely indicative of the apparent general belief that genitourinary surgery is something rather inconsequential and incidental. This portion of an otherwise excellent book should be wholly rewritten and a genitourinary surgeon should, at least, edit it.

---

**A Handbook of Practical Treatment.** In three volumes. By 79 eminent specialists. Edited by **John H. Musser,** M.D., Professor of Clinical Medicine, University of Pennsylvania; and **A. O. J. Kelly,** M.D., assistant Professor of Medicine, University of Pennsylvania. Volume I. Octavo of 909 pages. Illustrated. Philadelphia and London: W. B. Saunders Company. 1911. (Per volume, cloth, $6.00; half morocco, $7.50, net prices.)

This will prove a new, novel, important and valuable work when it is finally completed judging by the excellence of the first volume which has just been issued. The contributors who will develop the work under the editorial supervision of Dr. Musser will bring to it the best there is in medicine and surgery. In the list announced are the names of Roswell Park and Charles G. Stockton, of Buffalo, whose contributions will appear in the second volume.

The first part of the volume at hand is devoted to a discussion from a general viewpoint of the various therapeutic measures, the latter part to special treatment of the various general and local diseases. Physiologic and surgic therapeutics are considered in detail and an interesting and novel plan of presentation is that where the physician and surgeon are brought together in those borderland cases, which frequently prove so perplexing, in such manner that the reader is given the benefit of the discussion and the opinion of both internist and surgeon each from his own viewpoint.

Another interesting and valuable feature is that where slight ailments and symptomatic disorders are considered. Those minor complaints which wear the patient and monopolise the time of the general practitioner are given an unusual amount of space and the ill effects of failure to make complete and accurate diagnosis are emphatically pointed out. There is entire lack of multiplicity of treatments and methods and, for this reason, if for no other, it is effective and valuable.

**Modern Diagnosis and Treatment of Diseases of Children.** By **Herman B. Sheffield,** M.D., Instructor of Diseases of Children at the New York Post-Graduate Medical School. Octavo, pp. 619. Fully illustrated. Philadelphia: F. A. Davis Co. 1911. (Cloth, $4.50.)

A clinical treatise on the medical and surgical diseases of infancy and childhood, this volume embodies also the essentials of the theory of pediatrics so as to meet the needs of both the medical student and general practitioner.

In addition to less important innovations throughout the book, the author has ventured to introduce material changes especially in the following subjects: Feeble vitality of the newly born; hemorrhea, congenital and acquired; microcephalus; infant feeding; gastroenterocolitis; acute meningitis; tuberculosis and tetanism. Furthermore, special chapters are devoted to physical diagnosis and symptomatology, general and special therapeutics, congenital malformations, and mental diseases.

The work is based chiefly upon the author's very extensive experience in hospital, dispensary and private experience, hence is of the utmost practical value. The side notes are a great addition to the text.

The book is printed on heavy plate paper and we wish to mention in particular the illustrations all through the book, which are of such high class, mostly original, and some in colors. The treatise is so original in conception and design that it bespeaks the scientific skill and knowledge of its author from the first to the last page. The publishers, also, have done their work well.

**Case Histories in Pediatrics.** By **John Lovett Morse,** M.D., Assistant Professor of Pediatrics, Harvard Medical School. Octavo, pp. 314. Boston: W. M. Leonard. (Cloth, $3.00.)

The author has selected 100 actual case histories and classified them under section headings: Diseases of the new-born, of nutrition, specific infectious diseases, diseases of the blood, of the nervous system, and so on through the list. The book, taken as a whole, presents a post-graduate clinical course in diseases of children. Morse devotes much space and attention to diagnosis which is thoroughly discussed by naming diagnoses, which might with some reason be made and eliminating each in turn by careful references to clinical signs, terms and symptoms, the clinical terms being those which cannot be set aside and which all symptoms support.

There is a vitality in this kind of book that is refreshing. It deals with actual conditions as the physician meets them, and is a work to which he will look for helpful suggestion, as to a consultant, and not be disappointed.

---

**Atlas of Microscopic Diagnosis in Gynecology.** By **Dr. Rudolf Jolly,** Berlin. Translated by **P. W. Sheed,** M.D., New York. Royal octavo, pp. 192. With 52 lithographs in color and 2 textual figures. New York: Rebman Co. (Cloth, $5.50.)

The author lays great stress upon the microscopic examination of uterine scrapings and it is upon this that the atlas is based. It is profusely illustrated with excellent plates in colors. The descriptive text is simple of language and extremely clear in its explanations of the accompanying views of microscopic findings in various conditions. Methods of preparing and staining specimens for diagnosis are given in detail and much space has been devoted to a description of the technic necessary for good work and accurate results.

The atlas is published by Rebman which is merely another way of stating that letter press, plates and binding are without flaw.

---

**Memoranda on Poisons.** By **Thomas H. Tanner,** M.D. Eleventh revised edition by **Henry Leffmann,** M.D. Philadelphia: P. Blakiston's Son & Co. (Cloth, 75 cents.)

The necessity of reprinting another, the eleventh edition of this popular little manual, presented the opportunity for revision. The editor has inserted new matter introducing notes of several of the synthetics used as a substitute for morphin. The changes made in the former revision by the substitution of modern chemical nomenclature and the ommission of obsolete portions of the text seem to have met with general approval. The toxicology of poisonous food has been presented as fully as the concise character of the book allows.

---

**Golden Rules of Diagnosis and Treatment of Diseases.** By **Henry A. Cables,** B.S., M.D., Professor of Medicine and Clinical Medicine in the College of Physicians and Surgeons, St Louis. 12 mo, pp. 295. St. Louis: C. V. Mosby Co. 1911. (Cloth, $2.50.)

This is purely a reference work on diagnosis, treatment and remedial procedures and is of exceptional interest. A painstaking and searching examination of the literature of the subjects covered has been epitomised and to this has been added the results of the author's very extensive hospital experience. There is marked improvement in the method of construction of this book over that usually employed in works of its kind. The drugs mentioned are of use and one is not hampered by a multiplicity of measures from which he must pick and choose. It is almost impossible to mistake the importance of the suggestions contained in its pages for almost every paragraph begins with a black letter "Remember" which is impressive and suggestive. The book is one which will prove of great benefit.

